# Effect of forming degree in rotary hammer forging

**DOI:** 10.1038/s41598-026-41430-5

**Published:** 2026-03-30

**Authors:** Muhammad M. Hamdy

**Affiliations:** Department of Mechatronics Engineering, Misr University for Science and Technology (MUST), Sixth of October City, Giza Egypt

**Keywords:** Forging, Forming degree, Rotary forging, Rotary forging defects, Rotary hammer forging, Rotary press forging, Engineering, Materials science

## Abstract

Rotary press forging (RPF) has been introduced in the last century. Despite its advantages, it produces defects in the forgings such as mushrooming, eccentricity, and twisting. Rotary hammer forging (RHF) is a new process invented by the author to reduce such defects. RHF is considered as a multi-axes compression forging process where the material is subjected to several repeated hammering blows to be deformed incrementally and partially, while the produced deformation zone is swept over the whole area of the workpiece. Previous works showed that the specimen geometry, the inclination angle and the rotational speed affect such defects as mushrooming effect, eccentricity and twisting angle, but they are less severe in RHF than RPF. The present work has studied the effect of the forming degree (FD) on the forgings produced by both RPF and RHF to compare between the two processes. Special set-ups have been used where a die is rotating while either a pressing head or hammering head is used to deform the specimen. Independent variable parameters were chosen such that the specimen geometry H/D = 1, the inclination angle = 4$^{\circ }$, the rotational speed N =260 rpm, number of blows per revolution in case of RHF = 1.2. The results showed that FD has its influence on the mushrooming effect, twisting angle, and eccentricity, although they are less in the case of RHF. RHF reduces the defects referred to RPF by 5 to 13% for the mushrooming effect, 0 to 33% for the eccentricity, and 70 to 80% for the twisting angle. Thus, RHF is advantageous than RPF.

## Introduction

Conventional forging (CF) is extensively used as a manufacturing process in industry. The forming forces are applied either by continuous pressing in press forging (PF) or in the form of repetitive blows in hammer forging (HF). CF is a single axis compression (SAC) process since the upper and lower dies have the same axis. Rotary forging (RF) was first introduced in the early years of the 20th century^1^, and hereafter many rotary forging machines have been developed as reviewed by Standring and Appleton^2,3^.

**Fig. 1:**
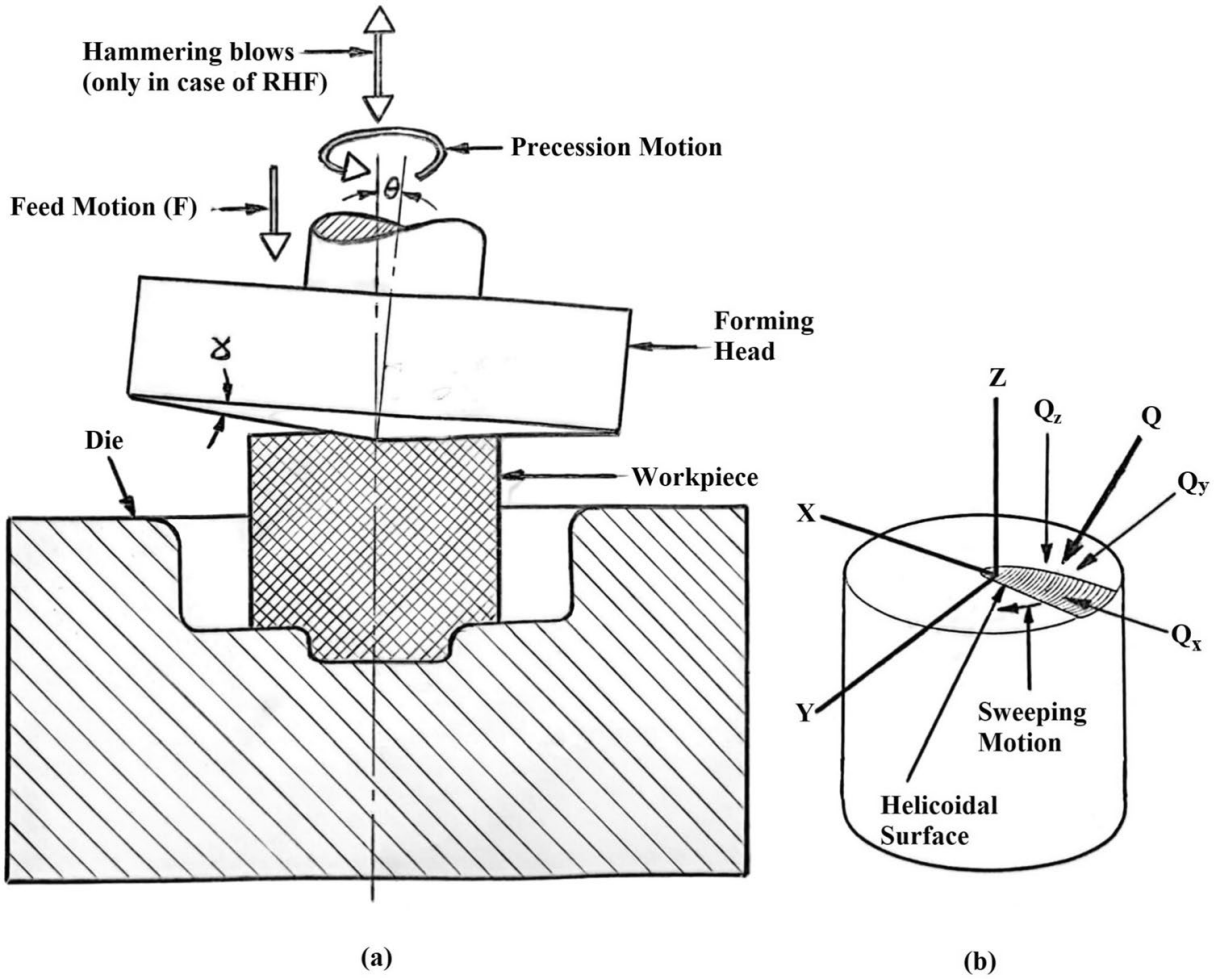
Rotary press and hammer forging processes: (**a**) Main elements and movements. (**b**) The indent and the forming force components.

RF machines have an inverted conical upper forming head of a wide angle with its apex in the center of workpiece top surface as shown in Fig. 1a. The axis of the cone is offset to the bottom die axis by a small angle $\Theta$, called the inclination angle. The die approaches the forming head by a continuous relative feed motion between the die and the forming conical head. The forming head produces an indent in the top surface of the workpiece as shown in Fig 1b. The projected area of the indent is about 10% of the whole area of the top surface of the workpiece. During the feed motion, the conical head is given a continuous rock-rolling motion to sweep the indentation over the surface of the workpiece until the final shape is achieved. Standring et al^4^ have divided the RF process into indentation phase and rotary or sweeping phase.

The author^5^ found it more precise to name RF as rotary press forging (RPF) since the die and the workpiece are in continuous contact as in conventional press forging. RPF has been extensively studied since 1970’s for its advantages and applications^2,6–8^. Although, RPF suffers from some disadvantages and defects such as mushrooming effect, eccentricity, and twisting^2,4,8–14^. The author^5,15^ has patented a new process, i.e. rotary hammer forging (RHF) to reduce such defects and disadvantages. RHF machine is nearly similar to RPF one. The only difference between them is that in RHF an oscillatory relative motion (in the form of hammering blows) is experienced by either the die or the indenter (forming head) as shown in Fig. 1a. The motion causes contact, and hence deformation (in the form of indent) over a small portion of the top surface of the workpiece. This contact is repeatedly gained and lost in each hammering blow. During hammering, a rock-rolling motion between the forming head and the die is experienced to sweep the indent over the workpiece top surface. Simultaneously, a continuous relative feed motion between the die and the indenter is applied to continue the process. Thus, RHF is described as a multi-axes compression process, where several repeated hammering blows are partially and incrementally deform the material, while the produced deformation zone is swept over the whole area of the workpiece. Thus, the only difference between RHF and RPF is that RHF is an interrupted (discrete) contact incremental deformation process, while RPF is a permanent (continuous) contact incremental one.

Abd-Eltwab et al.^16^ manufactured gear by RPF process. Their experimental work revealed that the blank size and the forming speed affect the tooth filling ratio and forming load. They found the appropriate dimensions of the blank for the best filling ratio. Jiang^17^ studied RPF of commercially pure titanium at temperature ranging from room temperature to 923 K. He stated that the deformation generates enough heat to cause dynamic recrystallization of the material. Due to the dynamic recrystallization, RPF enhanced the formability of the material and refined its grains.

The author has studied the characteristics of both RPF and RHF processes^5,18^. He found that the inclination angle and the specimen’s geometry affect the extruded length, the eccentricity, the mushrooming effect and the twisting angle. The defects of mushrooming, eccentricity and twisting were less severe in RHF than RPF. The author^18^ also studied the effect of the rotational speed on both RPF and RHF. He found that as the rotational speed increases, the mushrooming effect increases in RPF, but it is constant and smaller in RHF. Also, as the rotational speed increases, the twist angle increases in both RPF and RHF, although, it is smaller in RHF. The results showed also that there is no significant difference in the eccentricity between RPF and RHF. The eccentricity decreases sharply as the rotational speed increases in the range below 260 rpm. Above 260 rpm, the eccentricity in both RHF and RPF increases slightly as the rotational speed increases.

Forming degree (FD) is defined as the ratio (H-h)/H, where H and h are the original (initial) and final specimen heights respectively. Choice of the blank dimensions suitable to forge a product of certain volume affects the FD, the defects, and also the economics of the process. For example, for a blank of volume *V*, and final height after forging $= h$, there are two alternative cases:

- Case 1: Blank with large diameter $D_1$, and small height $H_1$, & $FD_1=(H_1-h)/H_1$
- Case 2: Blank with small diameter $D_2$, and large height $H_2$ & $FD_2 = (H_2-h)/H_2$

Thus $FD_1 < FD_2$. Han and Hua^19^ studied the effect of size of the cylindrical workpiece on the cold RPF using ABAQUS software. They took in their consideration the effect on metal flow and the mushrooming effect. The results show that increasing the initial diameter of the cylinder reduces the mushrooming effect. On the other hand, increasing the initial height of the cylinder increases the mushrooming effects. To date there is no published work on the effect of FD on RHF. Therefore, there is a need to study the effect of FD on RHF. Thus optimum value of FD could be suggested.

The aim of the present work is to know more about the characteristics of the RHF by studying the effect of the FD. Thus a comparison can be done between RPF and RHF.

## Experimental work:

**Fig. 2:**
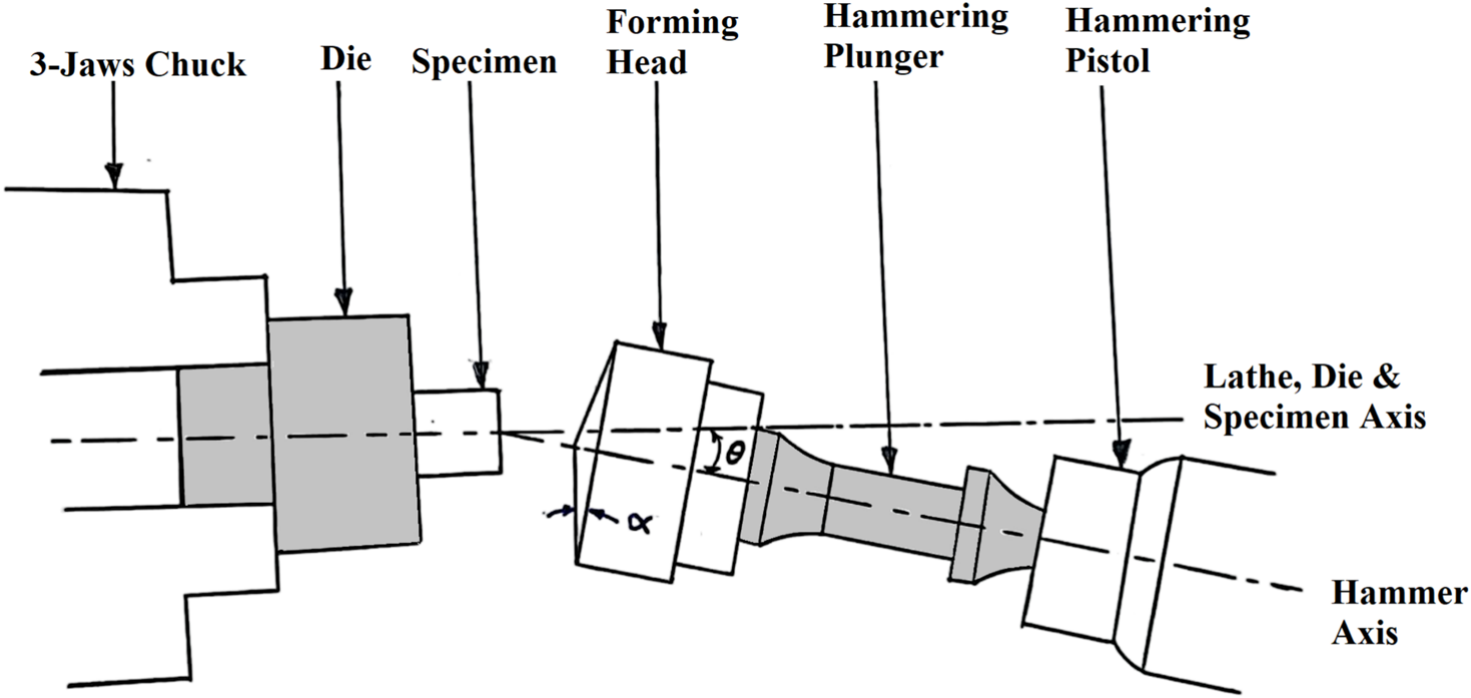
RHF set-up.

**Fig. 3:**
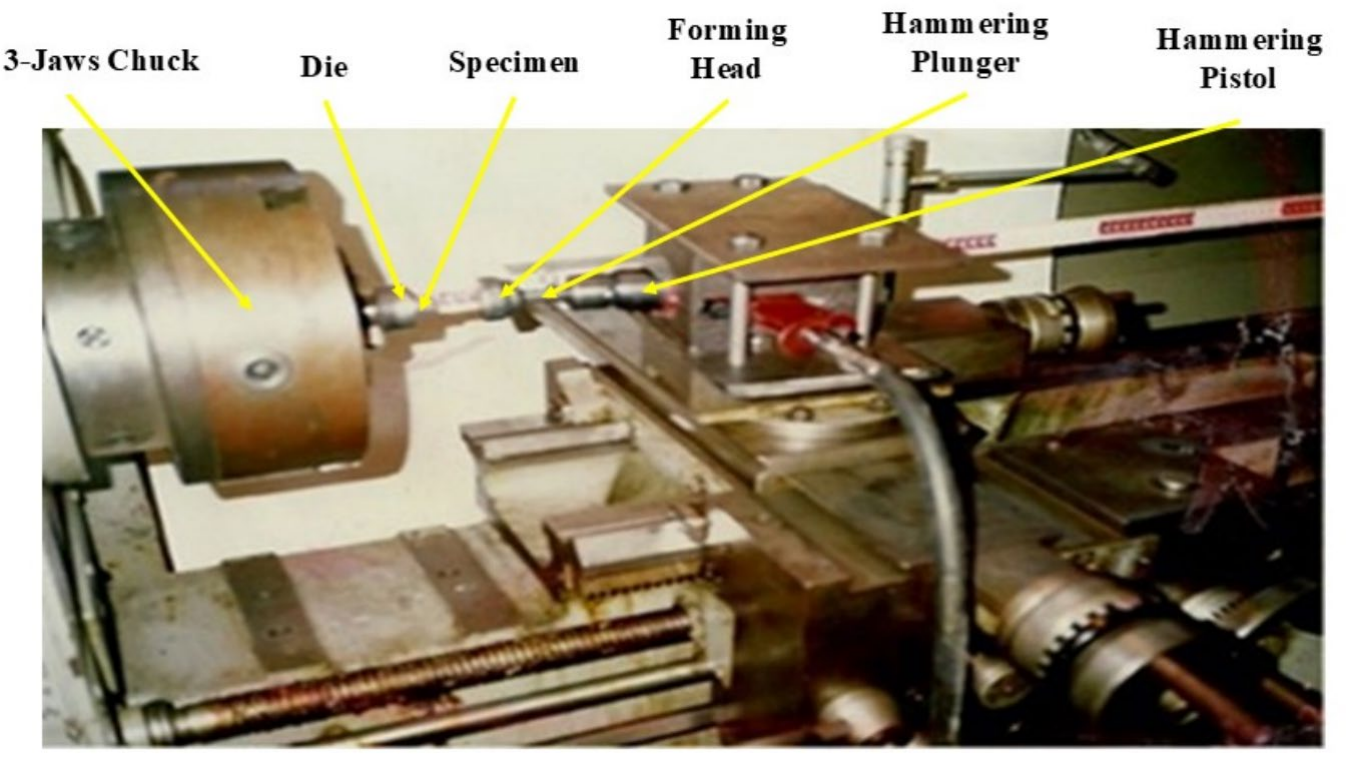
Close-up view of RHF set-up.

**Fig. 4:**
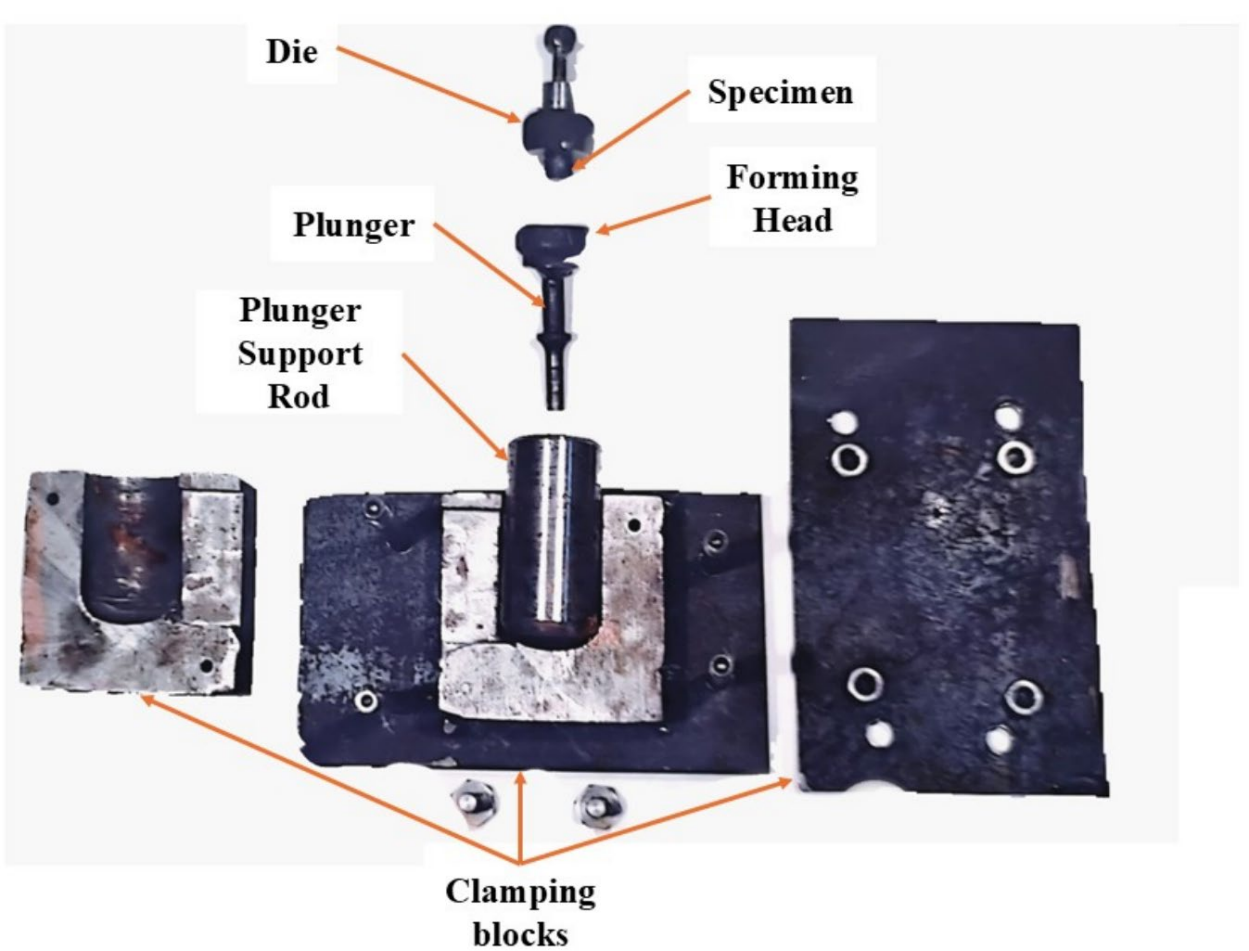
RPF set-up, dismantled.

An experimental set-up is given elsewhere^5^. Briefly, in RHF, the die is clamped in a centre lathe three jaws chuck and accommodates the specimen as shown in Figs. 2 and 3. A pneumatic hammering pistol is mounted on the compound rest of the lathe and fixes the conical forming head. The axis of the forming head inclines to the axis of the lathe, the die and the specimen. The precession is given by rotating the die about its axis, while the lathe carriage provides the required feed motion. In RPF set-up, the pneumatic hammering pistol is replaced by a steel rod as shown in Fig. 4. Although the cylindrical specimen was generally freely upset between the forming head and the die, it is worth mentioning that the specimen base is constrained in the same manner in both RPF and RHF experiments. Since this constraint is applied in both RPF and RHF, the comparison between them is fair to some extent.

RPF and RHF may be carried out as cold, warm, or hot working processes, each has its advantages and disadvantages, but they are extensively done at hot working conditions. The specimen’s material is a commercially pure lead due to its high ductility, and since forging it at room temperature is considered as a hot forming process. This is because dynamic recrystallization occurs during deformation at very low temperature as low as − 50 °C, or 223 K, i.e, far below room temperature. This means that forming lead at room temperature cannot be cold-worked, as it spontaneously self- anneals and recrystallizes. Tober et al.^20^ shows that lead metal is a good candidate for experimental modelling of hot working of different metals. It is worth mentioning that three specimens have been used for each testing condition.

The independent variable in the present work is the FD. Four ratios of FD have been chosen, i.e., 0.2, 0.4, 0.6 and 0.8. The other independent parameters are kept constant and chosen as follows:

- Specimen diameter (D) = 15 mm
- The specimen’s geometry (H/D) = 1
- The inclination angle ($\theta$) = the forming conical head angle ($\alpha$) = 4$^{\circ }$
- The feed (F) in case of RPF = the lathe carriage feed = 0.3 mm/rev
- The rotational speed (N) = 260 rpm
- The pneumatic pressure (P) for hammering pistol = 620 KPa
- Number of blows per revolution in case of RHF = 1.2

**Fig. 5:**
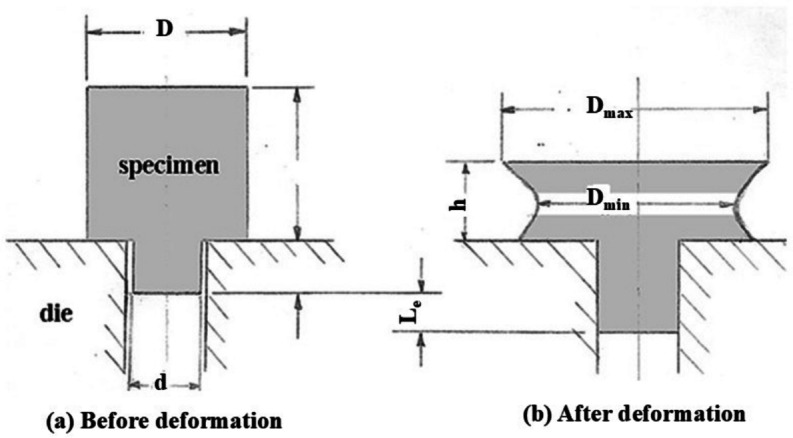
The Specimen, the Extruded Length & the Mushrooming Effect.

**Fig. 6:**
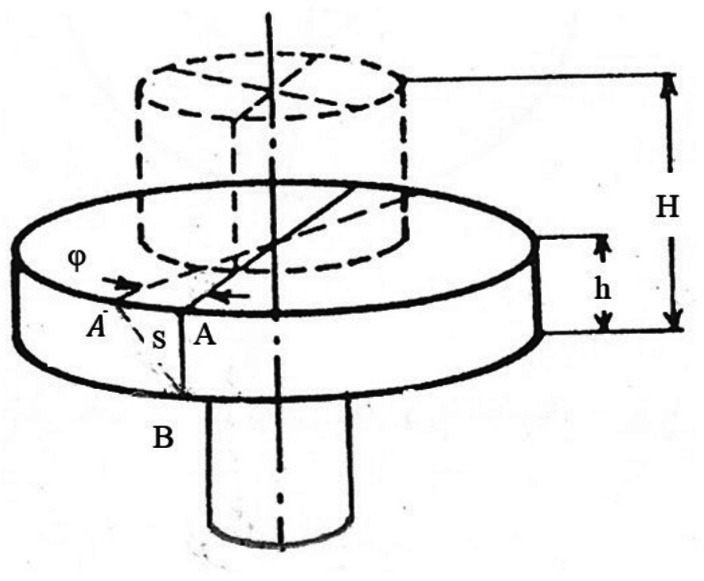
The twist angle.

**Fig. 7:**
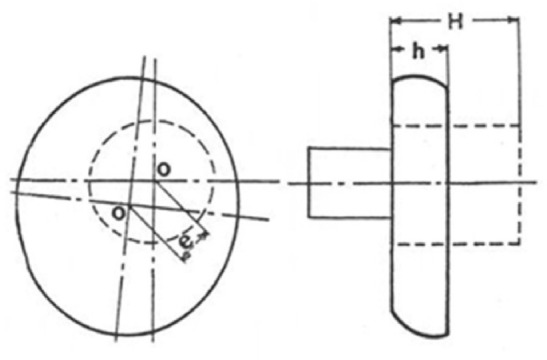
The twist angle.

The dependent variables studied in the present work are:

- The Extruded Length ($L_e$): As shown in Fig. 5, this is the length that has been extruded through the die opening.
- The Mushrooming Effect ($\psi$): This is defined as the ratio $D_{max}/D_{min}$, as illustrated in Fig. 5b.
- The Twist Angle ($\phi$): This is the rotary displacement of the top surface of the specimen relative to the bottom surface, shown in Fig. 6 by the movement of point *A* to $A'$. To measure the distance *S*, a line parallel to the specimen axis was shallowly scribed on the surface prior to forging. Twisting is indicated by the slope of this line, which was originally vertical.
- The Eccentricity (*e*): This is the amount of displacement of the new center of the specimen after forging from its original position, as shown in Fig. 7.

The extruded length, $D_{max}$, and $D_{min}$ were measured by a vernier caliper with 0.05 *mm* accuracy. The eccentricity was measured by a tool–room microscope with 0.01 *mm* accuracy. The twist angle was measured by a tool–room microscope with 0.1 degree accuracy.

## Results and discussions

In discussing the present results, it is important to consider that there is an angle of inclination *Theta* between the forming head axis and the specimen’s axis. Also, there is relative sweeping motion of the indent. Thus, the forming force “Q” has the following three components shown in Fig. 1:

$Q_x$ = the radial force

$Q_y$ = the sweeping force

$Q_z$ = the axial (indenting) force

Although the deformation mechanisms, and measuring forces, stresses and strains are helpful in explaining the results, they have not been considered in this work, and will be considered in future work. This is why some results and discussions are descriptive, showing the trend of different dependent variables or giving qualitative results.

Many works^21–26^ have studied the effects of applying vibrations during metal forming processes. The vibrations were produced by many ways such as ultrasonic or piezoelectric actuators. They found that applying vibrations has a beneficial effect on metal characteristics and reduces the forming loads. They attributed this to a reduction in friction force and flow stress, and the generation of localized heat due to the friction at the interface between the forming tool and the workpiece. This localized heat thermally softens the formed material. As a conceptual analogy, it is worth mentioning that RHF resembles to some extent vibrations-assisted forging, and may have the same effects.

The experimental results are given in Tables 1 and 2.

The reductions in the defects caused by RHF with respect to the defects of RPF are the criteria used to evaluate the superiority of RHF over RPF.

**Table 1:** RPF results.

Specimen No.	FD	$L_e$ (mm)	$\psi$	*e* (mm)	$\phi$
1	0.2	1.30	1.174	0.14	12.5
2		1.05	1.195	0.10	8.6
3		1.20	1.160	0.07	10.5
4	0.4	2.70	1.231	0.22	20.1
5		3.00	1.241	0.29	22.9
6		2.35	1.270	0.18	17.3
7	0.6	4.60	1.274	0.80	22.0
8		4.15	1.310	0.63	27.0
9		3.60	1.290	0.71	24.5
10	0.8	7.70	1.194	0.75	32.2
11		6.55	1.154	0.96	26.4
12		7.10	1.176	0.85	29.2

**Table 2:** RHF results.

Specimen No.	FD	$L_e$ (mm)	$\psi$	*e* (mm)	$\phi$
13	0.2	2.15	1.130	0.11	3.0
14		1.90	1.115	0.17	1.9
15		2.05	1.106	0.14	0.8
16	0.4	2.45	1.158	0.32	2.6
17		2.70	1.176	0.26	4.3
18		3.00	1.137	0.20	1.0
19	0.6	4.50	1.105	0.46	5.7
20		5.00	1.126	0.54	7.6
21		4.20	1.152	0.39	9.5
22	0.8	4.95	1.086	0.47	8.9
23		5.85	1.064	0.66	7.1
24		5.45	1.041	0.57	10.6

### Effect of FD on the extruded length

**Fig. 8:**
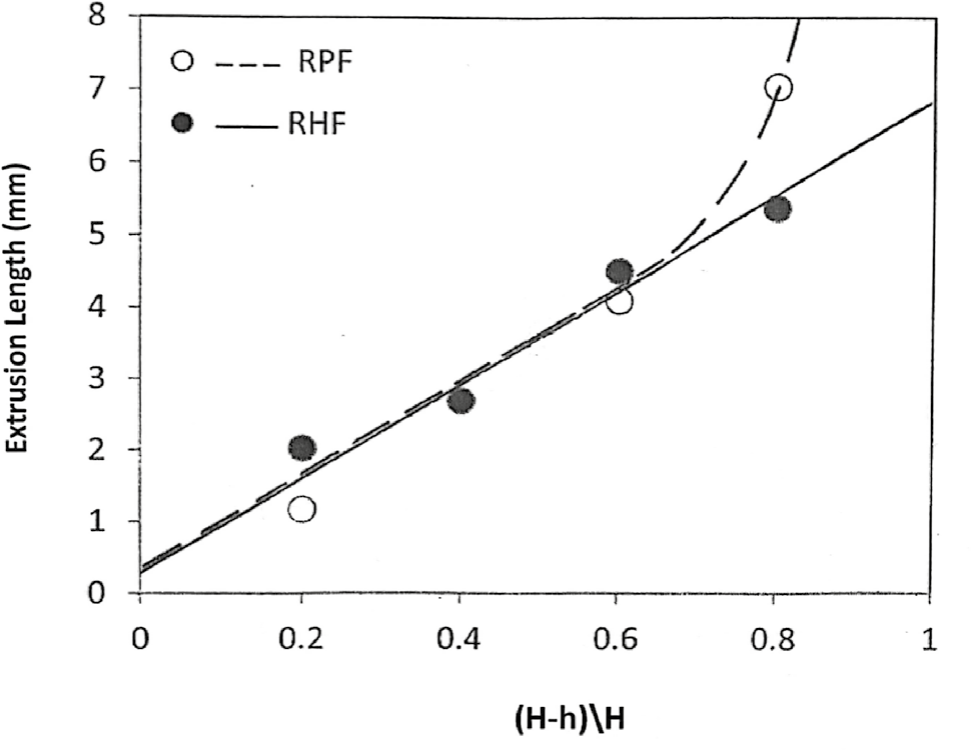
Effect of FD on the extruded length.

The results in Fig. 8 show that as the FD increases, the extruded length Le linearly increases in RHF up to 0.6. There is no significant difference in this behavior between RPF and RHF up to 0.6 FD. Above 0.6 FD, the extruded length continues to increase linearly in RHF, while it increases dramatically in RPF. This behavior is attributed to the increase in the volume of metal subjected to extrusion as the FD increases. These results agree with a previous work^27^.

### Effect of FD on the mushrooming effect $\psi$

**Fig. 9:**
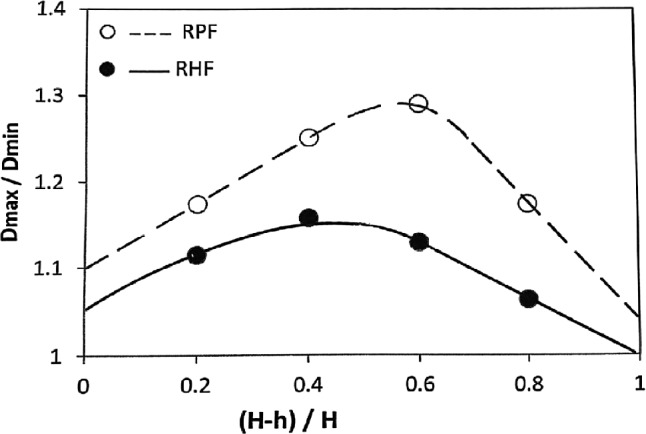
Effect of FD on mushrooming effect.

**Fig. 10:**
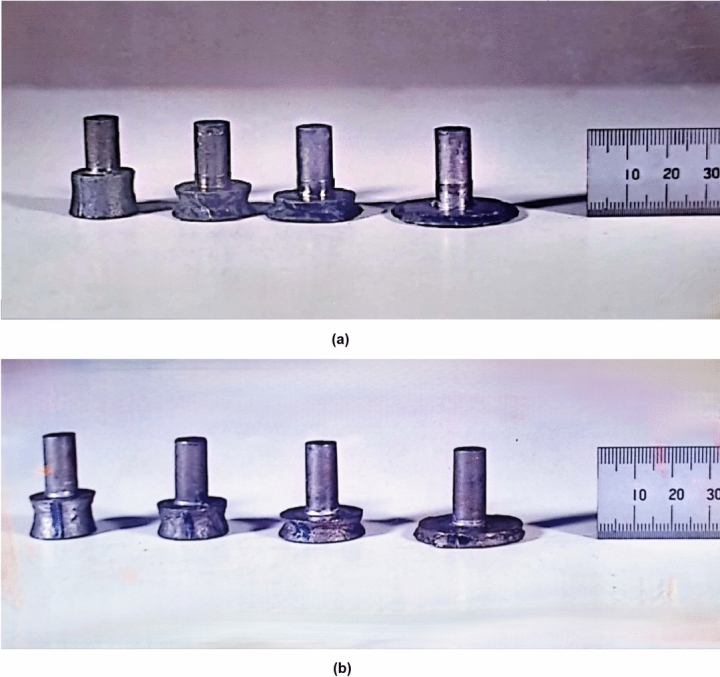
Photographs of Specimens (**a**) RHF; (**b**) RPF.

As shown in Figs. 9 and 10, the mushrooming effect $\psi$ has a maximum value at about 0.55 FD. This is true for both RHF and RPF, although $\psi$ is always higher in RPF. This is in good agreement with previous works^9,27–30^. As the FD moves away of 0.55, $\psi$ decreases. The mushrooming effect $\psi$ occurs when the shear strains inside the specimen are not uniform. Liu et al.^29^ have used a 3-D rigid-plastic finite element method to simulate RPF process to reveal the deformation mechanism of mushrooming effect. They attributed the mushrooming effect to the non-uniform deformation caused by the eccentric loading and the different forming degrees along the tangential direction at various heights of the workpiece. The peak in the curves is undesirable, but it appears that RHF produces a more uniform shape than RPF. Figure 9 shows that the improvement in the mushrooming effect due to RHF over that of RPF ranges from 5% far away from 0.55 FD up to 13% at 0.55 FD. This suggested that good choice of the FD far away from the peak value reduces the mushrooming effect, and hence, optimizes the uniformity of the workpiece shape. Such choice of FD is related to the choice of the initial blank dimensions.

### Effect of FD on the eccentricity

**Fig. 11:**
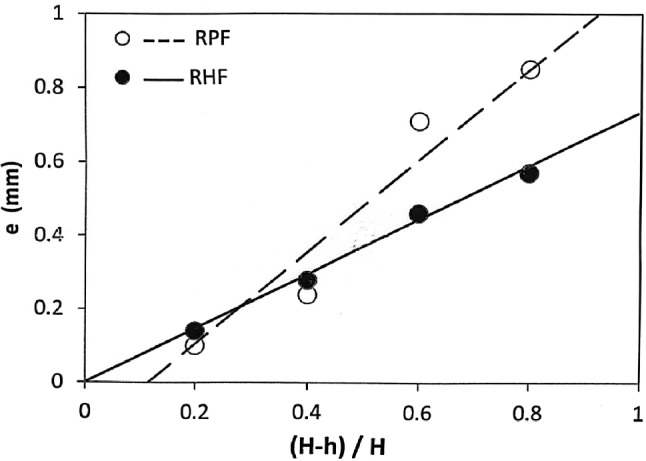
Effect of FD on the eccentricity.

It has been found that as the FD increases, the eccentricity increases linearly in both RHF and RPF as shown in Fig. 11. The eccentricity is always higher in RPF than RHF. Figure 11 shows that RHF gives a reduction in eccentricity with respect to that of RPF ranges from 0 at 0.2 FD up to 33% at 0.8 FD. This is in good agreement with previous works^3,4^ because in the case of large forming degree, there is a flow of large volume of metal, leading to higher eccentricity values. As the forming degree increases, specimen’s surface area increases. Thus, the ratio of the indentation area to the specimen’s surface area decreases. Standring et al^4^ stated that when the indentation is insufficient for the whole of the upper surface of the workpiece to be brought into contact with the indenter, radial flow occurs preferentially to one side, causing eccentricity in the workpiece. Hypothetically, it seems also that the difference in behavior between RHF and RPF is due to the difference in the value of the radial force $Q_x$.

### Effect of FD on the twist angle $\phi$

**Fig. 12:**
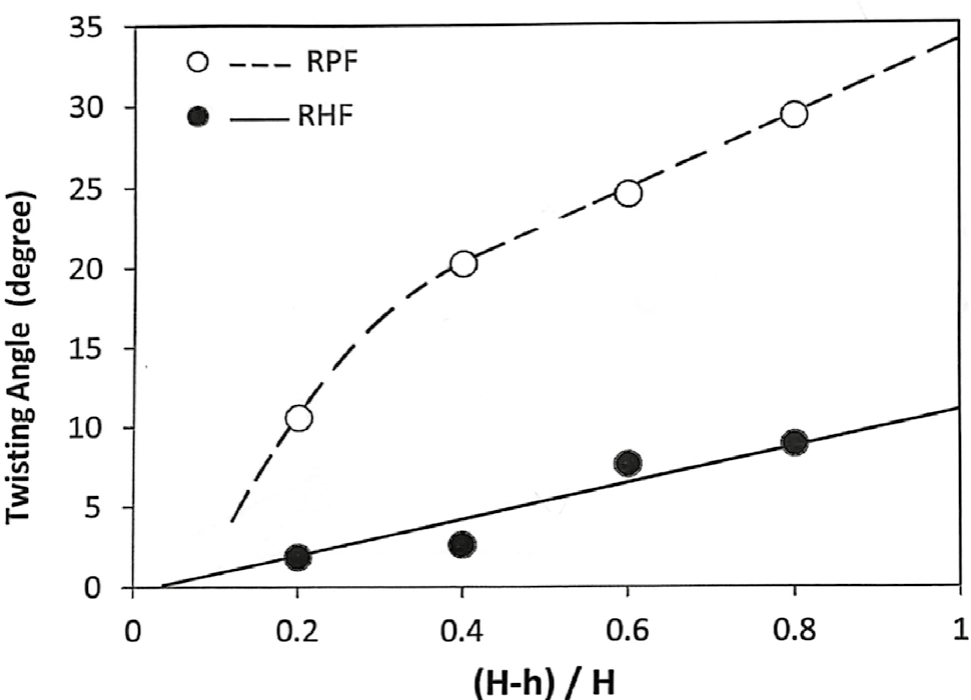
Effect of FD on the twist angle.

**Fig. 13:**
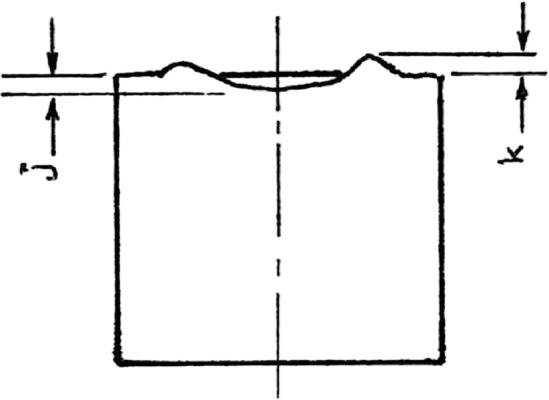
Elevation view of indented workpiece.

The results show that as the forming degree increases, the twist angle increases as shown in Figs. 10 and 12. This is in good agreement with previous works^3,31–33^. In the indentation phase of RF, an indent region is produced as shown in Fig. 13. This region comprises of penetration region of depth j and protrusion region of height k. As the indenting force $Q_z$ increases, j increases, and consequently, k increases. Qualitatively, it could be stated that the increase in j and k causes an increase in the sweeping force $Q_y$ and hence, causes an increase in the twist angle. Figs. 10 and 12 also show that the twist angle is always higher in RPF rather than in RHF. This is because $Q_y$ in Fig. 1b is partially effective in RHF due to the jump of the forming head over the leading edge of the protrusion shown in Fig. 13 during the noncontact phase of the stroke. This causes $Q_y$ to be zero in the noncontact phase. In case of RPF, $Q_y$ is continuously acting causing the higher values of the twist angle. Figure 12 shows that RHF gives a reduction in the twisting angle ranges from 80% at 0.2 FD up to 70% at 0.8 FD with respect to that of RPF.

## Conclusions

It could be concluded that:

1. The forming degree affects the extruded length, the mushrooming effect, the eccentricity, and the twist angle.
2. Defects of mushrooming, eccentricity, and twist angle are less severe in RHF than RPF.
3. The extruded length is less in RHF than in RPF.
4. RHF is advantageous than RPF, since RHF gives reductions with respect to the defect value caused by RPF as follows:
  - The deformation uniformity is achieved by reducing the mushrooming effect. Reduction in the mushrooming effect ranges from 5% to 13%.
  - Reduction in the eccentricity ranges from 0% to 33%.
  - Reduction in the twisting angle ranges from 70% to 80%.

## Data Availability

The datasets used and/or analysed during the current study available from the corresponding author on reasonable request.
